# Predicting adverse side effects of drugs

**DOI:** 10.1186/1471-2164-12-S5-S11

**Published:** 2011-12-23

**Authors:** Liang-Chin Huang, Xiaogang Wu, Jake Y Chen

**Affiliations:** 1 School of Informatics, Indiana University, Indianapolis, IN 46202, USA; 2 MedeoLinx, LLC, Indianapolis, IN 46280, USA

## Abstract

**Background:**

Studies of toxicity and unintended side effects can lead to improved drug safety and efficacy. One promising form of study comes from molecular systems biology in the form of "systems pharmacology". Systems pharmacology combines data from clinical observation and molecular biology. This approach is new, however, and there are few examples of how it can practically predict adverse reactions (ADRs) from an experimental drug with acceptable accuracy.

**Results:**

We have developed a new and practical computational framework to accurately predict ADRs of trial drugs. We combine clinical observation data with drug target data, protein-protein interaction (PPI) networks, and gene ontology (GO) annotations. We use cardiotoxicity, one of the major causes for drug withdrawals, as a case study to demonstrate the power of the framework. Our results show that an *in silico *model built on this framework can achieve a satisfactory cardiotoxicity ADR prediction performance (median AUC = 0.771, Accuracy = 0.675, Sensitivity = 0.632, and Specificity = 0.789). Our results also demonstrate the significance of incorporating prior knowledge, including gene networks and gene annotations, to improve future ADR assessments.

**Conclusions:**

Biomolecular network and gene annotation information can significantly improve the predictive accuracy of ADR of drugs under development. The use of PPI networks can increase prediction specificity and the use of GO annotations can increase prediction sensitivity. Using cardiotoxicity as an example, we are able to further identify cardiotoxicity-related proteins among drug target expanding PPI networks. The systems pharmacology approach that we developed in this study can be generally applicable to all future developmental drug ADR assessments and predictions.

## Background

Systematic and quantitative studies of adverse side effects have become increasingly important due to rising concerns about the cytotoxicity of drugs in development [1]. According to the US Food and Drug Administration (FDA), up to 90% of all experimental drug compounds going through clinical trials fail to gain FDA approvals due to problems such in efficacy, formulation, pharmacokinetics (PK), toxicology, or clinical safety. In the past decade, concerns over drug toxicity have risen significantly (from 10% to 20% during the decade), while concerns over drug efficacy have remained unchanged (25-30%) and concerns over drug PK have decreased significantly (from 40% to less than 10%). It is time for drug developers to design new and accurate models to assess unwanted side effects and drug actions before costly human clinical trials.

Recent research on the adverse side effects (ADR) [2] of drugs in cells has drawn attention to the inadequacy of the traditional "one drug, one target, and causal effect" model. Modern drugs are designed to regulate the functions of specific target proteins, or "drug targets". Efficacious drugs can break through human barriers of absorption, discretion, metabolism, and excretion to achieve desirable "on-target" effects. However, drugs may also bind to "off-target" proteins, potentially leading to unwanted side effects or ADRs, which range from mild drowsiness to deadly cardiotoxicity [3]. More appropriate models must be developed to take advantage of complex molecular responses of drugs in cells, by exploiting fully the relationships between chemical compounds, protein targets, and side effects observed at the physiological level [4]. The recent emergence of a systems approach to drug discovery has revitalized research in these areas of study [5,6]. By integrating the studies of human molecular networks, chemical compound similarity networks, and protein-drug association networks, systems biology researchers have spurred the development of systems pharmacology (also known as network pharmacology) [7][8]. By analyzing biological molecules and chemical entities in a variety of functional network contexts, drug developers can understand how drugs functions in a complex molecular system model [9], predict drug safety issues early [10,11], identify ADR events early [12,13], and design diagnostic tests for tailoring drug treatments to individuals [14].

Although the importance between systems biology and ADR had been recognized before, there had been no published report about how to practically predict ADR from molecular annotation data until this study [15]. Mutsumi Fukuzaki *et al. *used cooperative pathways and gene expression profiles to predict ADRs [12]. Nonetheless, large-scale validations and performances were not mentioned. Based on the concept of ADR similarity analysis [4], Nir Atias *et al. *applied canonical correlation analysis and a network-based diffusion to predict ADRs [16], with prediction precision at merely less than 0.5. Chemical structure-based approaches, also mentioned in Nir Atias' research, were used to predict off-targets [17] and ADRs [18]. While Andreas Bender *et al. *illustrated that this type of approach is quite sensitive in predicting ADR, the positive predictive value is generally quite low, averaging under 0.5 [19]. Moreover, protein-protein interaction (PPI) networks were not employed for predicting adverse drug interactions until Lucas Brouwers *et al. *presented the contribution of PPI networks to drug side-effect similarities [15]; however, the prediction precision was too low (= 0.298) to be of practical significance.

In this work, we propose a computational systems pharmacology framework consisting of statistical modeling and machine learning to predict ADR of drugs. Our framework is based on comprehensive integration of systems biology data, including drugs, protein targets, molecular annotation, and reported side effects. The contribution of our work is the following: First, drug-target interactions are expanded in global human PPI networks to build drug target expanding PPI networks. Second, drug targets are enriched by their gene ontology (GO) annotations to build drug target expanding GO networks. Third, ADR information for each drug is combined with drug target expanding PPI networks and drug target expanding GO networks. Fourth, statistics and machine learning are applied to build ADR classification/prediction models. Fifth, cross validation and feature selection are used to train prediction models. Without losing generality, we applied the framework to predict heart-related ADRs (i.e. drug cardiotoxicity), which are leading causes for clinical drug withdrawals [20]. The results of the cardiotoxicity prediction case study show that the performance of our approach (median AUC = 0.771) is significantly better than all previously-reported studies. The positive contribution of PPI networks (including both topological and biological information) and the GO annotations (including only biological information) for drug cardiotoxicity prediction are also quantified for the first time.

## Results

We report ADR prediction methods with cardiotoxicity as a case study. There are many ADRs related to cardiotoxicity, according to the index of the International Classification of Diseases 10th Revision (ICD-10) [21]. We merge all ADRs, each of which has an index ranging from I00 to I99 (classified as diseases of the circulatory system), into one group, *S_H_*. The ADRs related to cardiotoxicity in SIDER and their ICD-10 indices are listed in Table 1. In the ADR vs. drug target (expanding network) facts (See the framework introduced in the Methods section), if any one of *DS_nh _*is 1, where *D_n _*is drug *n*, and *S_h _*is in the group of heart-related ADR (see Table 1), then *DS_nH _*is set to 1; otherwise, *DS_nH _*is set to 0. The mathematical details are described in the Methods.

**Table 1:** The ADRs related to cardiotoxicity in SIDER and their ICD-10 indices

ADRs in SIDER	ICD-10 Index
Valvular Heart Disease	I08.8
Rheumatic Carditis	I09.9
Myocardial Infarction	I21
Myocardial Ischemia	I25.6
Heart Disease	I30-I52
Constrictive Pericarditis	I31.1
Pericardial Effusion	I31.3
Cardiac Tamponade	I31.9
Pericarditis	I32.8
Endocarditis	I39.8
Myocarditis	I40.8
Cardiomyopathy	I42
Second Degree Heart Block	I44.1
Complete Heart Block	I44.2
Heart Block	I45.5
Cardiac Arrest	I46
Sinus Tachycardia	I47
Tachycardia	I47
Junctional Tachycardia	I47.1
Multifocal Atrial Tachycardia	I47.1
Nodal Tachycardia	I47.1
Supraventricular Tachycardia	I47.1
Paroxysmal Ventricular Tachycardia	I47.2
Ventricular Tachycardia	I47.2
Heart Failure	I50
Congestive Heart Failure	I50.0
Right Heart Failure	I50.0
Cardiomegaly	I51.7
Cardiac Abnormality	I97.1

We evaluated the performance of ADR predictions in multiple experiments by applying standard statistical performance-evaluation measures, i.e., AUC (area under ROC curve), ACC (accuracy), SEN (sensitivity), and SPE (specificity). For each evaluation experiment, we repeated 10-fold cross validation three times and took median values to report prediction performances. To assess whether and how PPI networks (including both topological and biological information) or GO annotations (including only biological information) may contribute to a drug's cardiotoxicity-related ADR, we obtained results as described below.

### Use of biomolecular functional network data improves drug ADR predictions

We examined drug ADR prediction performance by integrating different sets of confidence-ranked PPI data derived from the HAPPI database [22]. The database contains comprehensive human functional and physical protein interaction/association data, at different confidence levels, from "1 Star" (low confidence, mostly functional association data) to "5 Star" (high confidence, mostly physical interaction data).

**Figure 1:**
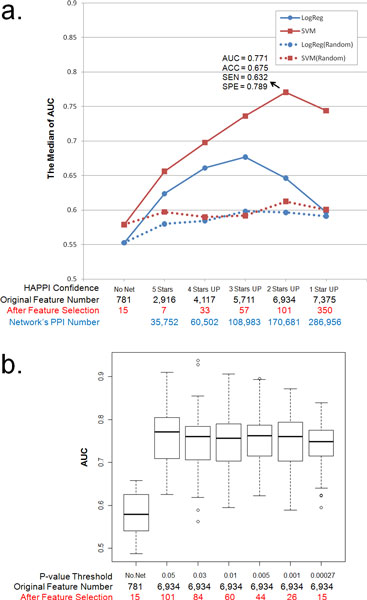
**The performances of SVM and logistic regression in the different confidence levels of PPIs**. a) "No Net" means the prediction models used ADR vs. drug target facts; "5 Stars" means we used PPIs in HAPPI under the confidence of 5 stars level; "4 Stars UP" means we used PPIs in HAPPI under the confidence of 4 to 5 stars level; and so forth. A red solid line represents the performance with SVM, while a blue solid line shows the performance with logistic regression. A red dotted line means we expanded the drug target network by replacing the PPI networks with random networks as control experiments or a base line of the performances with SVM, while a blue dotted line illustrates the effect of using logistic regression. AUC: area under ROC curve. ACC: accuracy. SEN: sensitivity. SPE: specificity. b) This box plot comparisons performances from using "2 Stars UP" PPI-expanding networks versus SVM under different *p*-value criteria in the feature selection.

We can observe significant contributions of PPI networks to both prediction models, as shown in Figure 1(a). When SVM (red solid line) is applied, the performance prediction goes up from AUC = 0.579 (using "No Net", or not PPI network data) to AUC = 0.771 (using "2 Stars UP" PPI network data). The use of PPI data bring up prediction performances significantly, i.e., Accuracy = 0.675, Sensitivity = 0.632, and Specificity = 0.789. The increased AUC of the "2 Stars UP" condition over the "No Net" condition is very significant, with *p*-value = 4.93e-35 based on the *t*-test. If one further includes the lowest confidence level ("1-Star" PPI network data) into the drug target expanding network, the prediction performance decreases slightly due to noise in molecular networks. The performance curve of logistic regression (blue solid line) is comparable to, yet systematically lower than, that of SVM, moving up from AUC = 0.553 (using "No Net") to AUC = 0.677 (using "3 Stars UP" PPI network data). The performance of "3 Stars UP" PPI network data is lower than that of "2 Stars UP" PPI network data, at Accuracy = 0.649, Sensitivity = 0.564 and Specificity = 0.789. The increased AUC of the "3 Stars UP" condition over the "No Net" condition is also very significant, with *p*-value = 6.83e-18 based on the *t*-test. However, the decreased AUC performance between "3 Stars UP" condition over the "2 Stars UP" condition is also noticeable, likely due to the functional nature (no longer biased towards physical PPI events) of biomolecular networks at the "2 Stars" level reported by the HAPPI database.

In order to control for the effects of using any types of (random) biomolecular networks and their possible contributions to ADR predictions, we also tested the model's performance with the use of randomized PPI networks which shared the same network topologies as actual PPI networks. Figure 1(a) shows that the performance curves using random networks slightly increased (with AUC > 0.55), when SVM (red dotted line) and logistic regression (blue dotted line) were applied. This result occurs because the original relationships between drugs and drug targets are still retained in the simulated random PPI networks. The additional gained prediction power, however, can only be explained by the embedded useful network information that our prediction model automatically learned from real biological network structures. These results strongly suggest that the contribution of PPI network data to drug ADR prediction is primarily due to useful functional information embedded in biomolecular functional association networks of drug targets and their related proteins, whereas network topology alone only plays a peripheral role.

We also studied whether the increase in our model's prediction performance may be due to the increase in the total number of features when PPI network data are introduced. For this purpose, we focus on the result obtained from the use of "5 Stars" PPI network data, in which the number of features obtained by the prediction models becomes much smaller than that without using any network information. We noted that the AUC of this experimental result is better than that without using any network information (*p*-value = 2.70e-8 and 8.22e-9 for *T*-test, when we used SVM and logistic regression, respectively). To further confirm the relationship between the number of features captured in the model and the model performance, we performed another experiment in which we gradually decreased feature number "2 Stars UP" PPI data in the SVM prediction model by lowering feature selection thresholds. Figure 1(b) showed that there is no significant (*p*-value = 0.469 using ANOVA) decrease of prediction performances, when the number of features is filtered down. These observations further support our original finding that the contribution of PPI network for a drug's ADR prediction performance primarily comes from network data themselves.

### Integration of GO annotations also improves drug ADR predictions

**Figure 2:**
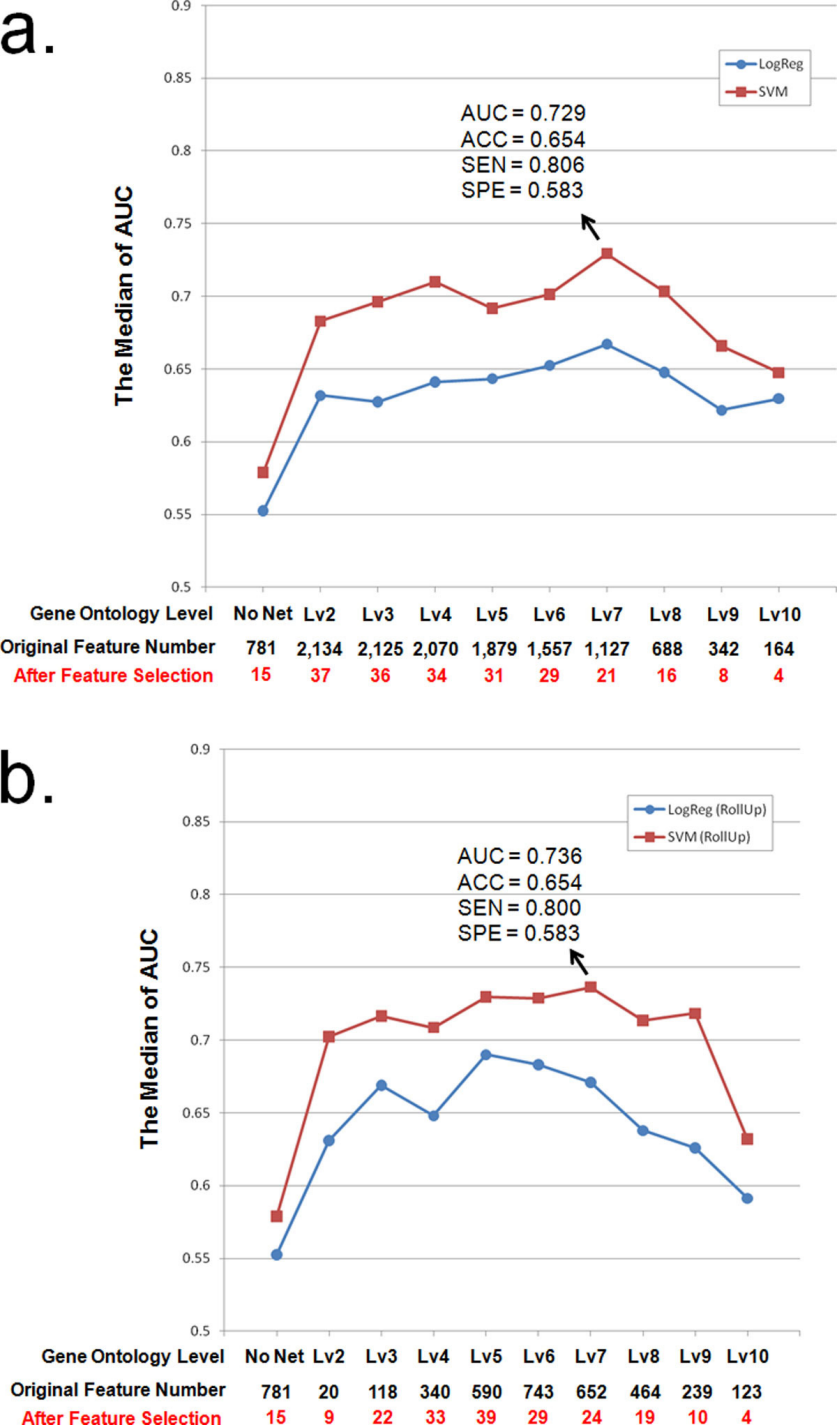
**The comparison of performances of prediction models including drug targets' GO term annotation**. a) This broken line graph illustrates the performances when the prediction models used different levels of GO term annotations. A red line represents results using SVM, while a blue means represents results using logistic regression. The x-axis shows the different threshold levels of the GO terms. "No Net" means the prediction models used ADR vs. drug target facts; "LvN" means we just used a GO term equal to or deeper than level N in the GO hierarchy (we defined the term of "biological process" as level 1). b) The x-axis shows each level of the GO terms. "LvN" means we replaced the GO terms deeper than level N with their level N ancestors.

We also examined drug ADR prediction performance by integrating GO annotations available for each drug's protein targets. In two experiments (Figure 2), we directly incorporated into our prediction models GO annotation labels of drug target proteins. Since each protein-coding gene may be annotated by many GO terms from different GO hierarchical levels, we carefully designed experiments to eliminate potential ADR prediction performance biases due to non-uniformity of GO term hierarchical levels. Therefore, we show in Figure 2 how GO terms aggregated to different GO hierarchical levels can contribute to prediction results based on different thresholds, the number of GO terms satisfying each threshold, and the number of GO terms selected into each model. Since GO hierarchical level = 1 is not biologically meaningful and there is insufficient data for GO hierarchical levels from 11 to 15, results for these categories are not shown.

In the first experiment (Figure 2(a)), the GO terms equal to or deeper than specified threshold GO hierarchical levels are used to annotate drug targets for comparative drug ADR prediction performance analysis. Our results suggest that the prediction performances with the use of GO terms, regardless which predictive modeling method is used and which criteria is used for comparisons, are always better than those without the use of GO terms. In particular, when GO term level 7 (Lv7) is chosen, a best performance can be achieved with the use of SVM, in which we observed AUC = 0.729 and Sensitivity = 0.806; in comparison, "No Net" (without the use of GO term information) has AUC = 0.579. The improvement in overall ADR prediction performance defined by AUC is significant (*p*-value = 1.80e-18, based on *t*-test).

In the second experiment (Figure 2(b)), the GO terms deeper than level N are replaced by their level N GO term ancestors to annotate drug targets for comparative drug ADR prediction performance analysis. We call this process a "Roll Up" and observed similar results as in the first experiment. In particular, when GO term Lv7 is chosen, a best performance can be achieved with the use of SVM, in which we observed AUC = 0.736 and Sensitivity = 0.800. The improvement in overall ADR prediction performance defined by AUC over the "No Net" experiment is also determined to be statistically significant (*p*-value = 7.75e-17, based on *t*-test).

Based on the above two experiments using GO terms, we can make the following conclusions. First, the use of GO annotations can help improve a drug's overall ADR prediction performance. Drug ADR prediction performances achieved with the best use of GO annotation (AUC = 0.736) are almost comparable to those achieved with the best use of PPI networks (AUC = 0.771). Second, SVM models can help achieve better performance than logistic regression model can based on our case studies. Third, to achieve the best ADR prediction performance, it is best to choose SVM models and use GO biological process categorical terms at sufficiently detailed term levels (e.g., level 7) to annotate drug targets. Fourth, by evaluating detailed prediction performances achieved with PPI networks (SEN = 0.632, SPE = 0.789) and GO annotations (SEN = 0.800, SPE = 0.583), we discovered that integration of biomolecular network data can increase the specificity (SPE) of ADR predictions, while the integration of GO annotation data can increase the sensitivity (SEN) of ADR predictions.

### A good ADR prediction model is concentrated not only on drug targets implicated with the ADR events, but also on many non-target proteins directly linked to ADR mechanisms

**Figure 3:**
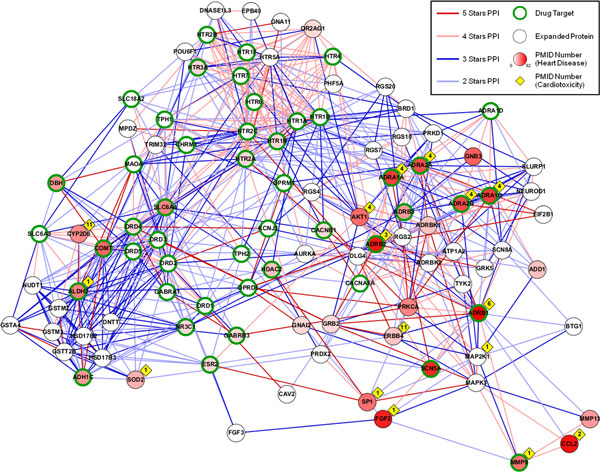
**Cardiotoxicity-associated PPI network**. The 101 proteins that passed the feature selection in the "2 Stars UP" PPI network are used to establish a cardiotoxicity-associated PPI network. We use light blue, deep blue, light red, and deep red lines to represent 2 to 5 stars PPIs respectively. A node with a green and thick border means a drug target which is docked by a drug reported to have cardiotoxicity-associated ADRs. Node color illustrates the paper number we obtained from PubMed by searching the protein name and the term of "Heart Disease" together. The diamond shape on the top right site of a node shows the paper number we obtained from PubMed by searching the protein name and the term of "Cardiotoxicity" together.

We examined further the biological network contexts for 101 proteins selected automatically by the SVM prediction model as features. We expanded these "seed proteins" with "2 Stars UP" PPI interactions to build a PPI interaction network using the nearest neighborhood expansion method [23]. In Figure 3, we used node color and counts (in diamond shapes) to show how much evidence from PubMed might be identified in each protein.

Many selected proteins were found to have close relationships to cardiotoxicity. For example, ADRB1 (Adrenergic, beta-1-, receptor) mediates hormone epinephrine and neurotransmitter norepinephrine. The polymorphisms of ADRB1 have been shown to be involved in drug cardiotoxicity in heart failure [24]. Autoantibodies against the beta-1-adrenergic receptor have also been shown to have idiopathic dilated cardiomyopathy in some patients [25-27]. Therefore, it gives us great comfort that ADRB1 as a known drug target is also a part of the predictor.

We also observed that the drug target expanding network can bring forth additional cardiotoxicity-related non-target proteins, e.g., ERBB4 and CYP2D6. ERBB4, a v-erb-a erythroblastic leukemia viral oncogene homolog 4, is a member of the type I receptor tyrosine kinase subfamily and encodes a receptor for NDF/heregulin. Targeted deletion and inhibition of ERBB4 signaling may lead to congestive heart failure resulting from cardiovascular defects [28,29]. CYP2D6 encodes a subunit of the cytochrome P450 superfamily of enzymes. The gene is specifically expressed in the right ventricle and its genetic polymorphism is known to be associated with cardiotoxicity, including a patient's poor anti-arrhythmic activity, severe cardiovascular, or dilated cardiomyopathy [30,31].

Using all GO annotations from GO term level 7 "Rollups" to build predictors with the SVM model, we developed a final list of 24 GO terms selected into the best cardiotoxicity prediction model (See Figure 2(b)). In Table 2, we list the 24 GO terms and their IDs. Interestingly, many of these terms appear to be related to heart disease or cardiotoxicity. There are many known literature reports linking these terms to cardiotoxicity. For example, Avkiran *et al. *[32] described the MAPKKK signaling cascades in heart failure; Yatani *et al. *[33] showed G proteins' roles in heart rate regulation; Plunkett *et al. *[34] examined the role of dopamine receptor on the cardiovascular system; the GO terms of GO:0008016 and GO:0045823 are defined as the terms related to regulation of heart contraction; GO:0051924 is involved in the regulation of calcium ion transport, a critical process in cardiovascular functions; Gosalvez *et al. *[35] showed evidence of the link between potassium transport and cardiotoxicity; and so on. Apparently, the prediction model works by integrating biologically significant drug targets within known cardiovascular side effects and the related non-target protein functional partners implied in cardiovascular functions or cardiotoxicity.

**Table 2:** GO terms that passed the feature selection in the replaced level 7 terms

ID	GO Term
GO:0000165	MAPKKK Cascade
GO:0002031	G-Protein Coupled Receptor Internalization
GO:0007194	Negative Regulation of Adenylate Cyclase Activity
GO:0007210	Serotonin Receptor Signaling Pathway
GO:0007212	Dopamine Receptor Signaling Pathway
GO:0007612	Learning
GO:0007613	Memory
GO:0008016	Regulation of Heart Contraction
GO:0009123	Nucleoside Monophosphate Metabolic Process
GO:0019935	Cyclic-Nucleotide-Mediated Signaling
GO:0043268	Positive Regulation of Potassium Ion Transport
GO:0043278	Response to Morphine
GO:0043408	Regulation of MAPKKK Cascade
GO:0045762	Positive Regulation of Adenylate Cyclase Activity
GO:0045823	Positive Regulation of Heart Contraction
GO:0045859	Regulation of Protein Kinase Activity
GO:0045893	Positive Regulation of Transcription, DNA-Dependent
GO:0046488	Phosphatidylinositol Metabolic Process
GO:0051924	Regulation of Calcium Ion Transport
GO:0051932	Synaptic Transmission, Gabaergic
GO:0051937	Catecholamine Transport
GO:0051969	Regulation of Transmission of Nerve Impulse
GO:0072511	Divalent Inorganic Cation Transport
GO:2000147	Positive Regulation of Cell Motility

## Discussion

In this work, we not only built effective ADR prediction models, but also showed that the use of biomolecular networks or gene annotations may independently improve ADR prediction performances. Integrating gene network and gene annotation allows the use of deeper level of biological knowledge [36] to increase a model's prediction performance. In future work, it would be preferable to study functional relationships between proteins that are not directly associated. Additional experimentally-based genotype-phenotype information, e.g., those derived from genome-wide association studies, may also be useful, as several recent studies of genetic polymorphisms of cardiotoxicity-inducing enzymes have already showed [37,38].

We developed a general conceptual framework and demonstrated how to build practical ADR prediction models, using cardiotoxicity as a case study. For other drug ADR predictions, hepatotoxicity and nephrotoxicity are also critical issues to consider in drug development. Decision trees were developed and trained by others to predict hepatotoxicity and nephrotoxicity [39], relying on a drug's chemical properties to predict ADRs. Therefore, we plan to soon assess hepatotoxicity and nephrotoxicity in a future extension of this work and compare our performances with those of other approaches.

## Conclusion

In this study, we presented a systems pharmacology framework for predicting drugs' ADR, using cardiotoxicity as an example. Our method is based on SVM and logistic regressions, by integrating ADR information, drug-target data, PPI networks, and GO term annotations. Our results indicated that integrating functional biomolecular association networks or detailed GO annotation could significantly improve a drug's ADR prediction. Particularly, comprehensive functional biomolecular association networks are shown to be useful for increasing specificity, while detailed gene annotation information are shown to be useful for increasing sensitivity. Moreover, proteins used to automatically build the prediction models are shown to further reveal related biological functions for cardiovascular health and cardiotoxicity. Overall, our study described a novel way of predicting ADRs, with comprehensive incorporation of additional prior knowledge ADR assessments.

## Methods

**Figure 4:**
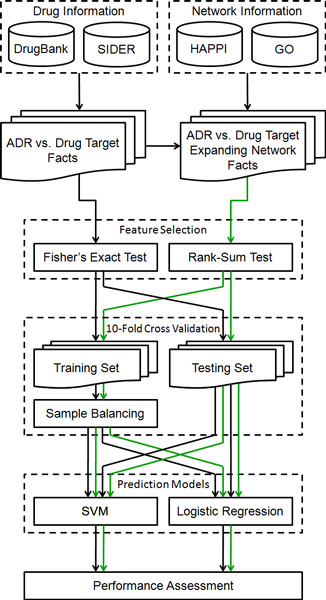
**A framework for adverse drug reaction prediction**. Black arrows indicate data flows for ADR vs. drug target facts. Green arrows indicate data flows for ADR vs. drug target expanding network facts. (DrugBank: Drug-Target data; SIDER: Side Effect Resource; HAPPI: Human Annotated and Predicted Protein Interaction; GO: Gene Ontology; ADR: adverse drug reaction; SVM: support vector machine; Facts: multi-dimensional datasets)

A framework for ADR prediction is shown in Figure 4, which includes drug and network information retrieval, feature selection, cross validation, sample balancing, prediction models, and performance assessment. There are two types of data flows in the flowchart: 1) Black arrows indicate data flows for ADR vs. drug target facts. 2) Green arrows indicate data flows for ADR vs. drug target expanding network facts, which are generated by integrating ADR vs. drug target facts and network information.

### Drug and network information retrieval

We used three major public databases in our study.

First, the DrugBank database is exploited as a bioinformatics and cheminformatics resource, which contains drug and drug target information [40]. Up to May 2011, there were 5,461 drugs and 3,880 proteins, which formed 13,457 unique drug-target pairs in DrugBank, and they were extracted as main drug target information in this study.

Second, the Side Effect Resource (SIDER) database is also involved. This database aggregates FDA drug labels and disperses public information on ADRs [41]. There were 877 drugs, 1,447 kinds of ADR, and 61,824 relationships among drugs and ADRs obtained from COSTART and Euphoria-related ADRs in SIDER. There are 578 drugs overlapped between DrugBank and SIDER.

Third, the Human Annotated and Predicted Protein Interactions (HAPPI) database [22] is used as a global human PPI resource. HAPPI integrates HPRD, BIND, MINT, STRING, and OPHID. Most importantly, HAPPI provides a confidence star quality rating from 1 to 5 for each interaction based on the initial data sources, data generation methods, and number of literature references for the interaction. Excluding self PPIs, there are 116,275 PPIs, 61,698 PPIs, 48,481 PPIs, 24,750 PPIs, and 35,752 PPIs involved in the data set from 1 star to 5 stars, respectively. This data can be used to expand the network of drug targets.

Finally, the Gene Ontology (GO) project provides hierarchical terms, including biological processes, cellular components, and molecular functions, to describe the characteristics and annotations of gene product [42]. In this study, we only use biological processes, from a general term "biological process" in level 1 to specific terms in level 15, to expand the features in the prediction models from drug targets to the GO terms in order to investigate the biological meanings between drug targets and ADRs. There are 3,715 biological process terms utilized for annotating the drug targets in this study.

### ADR vs. drug target (expanding network) facts

**Figure 5:**
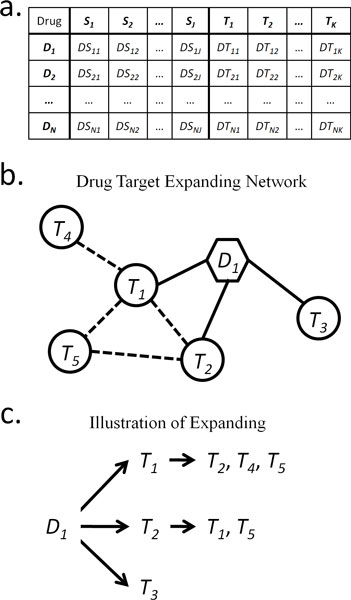
**Format of ADR vs. drug target facts and an example of drug target expanding network**. a) This figure shows the format of ADR vs. drug target facts. *D_n_*, *S_i_*, and *T_j _*mean drug *n*, ADR *i*, and target *j *respectively. b) This figure is an example of a drug target expanding network. The dotted lines mean PPIs. c) This part illustrates the process of the drug target expanding one level in PPI network. *T_1_*, *T_2_*, and *T_5 _*are present twice in all branches of this expanding "tree", thus the values in cells *DT_11_*, *DT_12_*, and *DT_15 _*in ADR vs. drug target facts are all 2; the rest, including *DT_13 _*and *DT_14_*, are both 1.

By combining the drug target information in DrugBank with the ADR information in SIDER, we obtained the ADR vs. drug target facts. The facts follow the format shown in Figure 5(a). If drug *n *has a side effect *j*, the value in cell *DS_nj _*(*n *= 1...*N*, and *j *= 1...*J*) at the intersection of column *S_j _*and row *D_n _*is 1 or "TRUE"; otherwise, it is 0 or "FALSE". So does the value in cell *DT_nk _*(*n *= 1...*N*, and *k *= 1...*K*) at the intersection of column *T_k _*and row *D_n _*if drug *n *docks to drug target *k*. The binary data *DS_nj _*and *DT_nk_*, representing the ADR vs. drug target facts, can be then used for prediction model training and testing: each ADR *S_j _*is prediction output (response variable) and targets from *T*_1 _to *T_K _*are features (dependent variables).

When the drug targets expand one level in a PPI network or are annotated by using the GO terms, the value in cell *DT_nk _*will be integer instead of binary, because the association between drug *n *and drug target *k *could be repeatedly present in the drug target expanding network. Figure 5b shows an example of a drug target expanding network, and Figure 5c shows the drug target expanding process and the repeated presences of *T_1_*, *T_2_*, and *T_5_*. The repeat number here can be regarded as the weight of the relationship between drug and target under network level; moreover, this weight can be also used to study the contribution of each target to ADRs.

### Feature selection

Since thousands of features (drug targets) are required to build prediction models, this process is exhaustive and memory consuming. Moreover, some statistics tools, such as R, have memory limitations [43]. Hence, it is necessarily to filter out the features that would make little contribution to the response variable. If the data type of cell *DT_nk _*is binary, Fisher's exact test will be used; otherwise, Wilcoxon rank-sum test will be used. In both methods, features will be selected if their *p*-values are smaller than 0.05.

### Sample balancing

The sample sizes of output classes are usually bias and imbalance, especially in medical data [44]. Consequently, the accuracy of the prediction result would be overestimated. In order to prevent this problem from happening, sample balancing method is also applied in this study. First, we randomly separate the major class into many parts. Each part contains a sample size close to that of the minor class. Second, we combine every part of the major class with the minor class as training sets. The input data is separated into ten parts in the process of 10-fold cross validation: nine parts are taken to do the sample balancing and the remaining one is used to validate the prediction models. The training sets are balanced, while the validation set is still imbalanced in the sample sizes of classes, so the performance will be more reliable.

### Prediction models

For comparisons, the prediction models in this study include two independent procedures: 1) machine learning - support vector machines (SVM), and 2) statistical modeling - logistic regression. A SVM package of R, called "e1071" [45] is used in this study. For kernel functions, we choose a nonlinear one: Gaussian radial basis function, which is also the optimized kernel function. This SVM package provides fitted probabilities numerically from 0 to 1, and so does the logistic regression package used in this study, named as "generalized linear models" [46].

## Competing interests

JYC and XW are co-founders of Medeolinx, LLC. This academic and private sector collaboration leverages Medeolinx's intellectual property, technologies, and equipment. However, this work does not promote any Medeolinx-related products or services.

## Authors' contributions

JYC conceived this work, guided the research team by providing ideas and feedback along the way, and revised the manuscript. LH integrated the information in public datasets, developed the analysis workflow, chose classifiers, and assessed the performance of the analysis. XW introduced SIDER to this study, specified mathematical descriptions, refined results, and helped improve the readability of the manuscript. All authors proofread and approved the final manuscript.
